# Utilizing glauconite extracts to enhance soil health and sugar beet (*Beta Vulgaris* L.) performance in salt-affected soil

**DOI:** 10.1371/journal.pone.0337492

**Published:** 2026-02-23

**Authors:** Mahmoud M. A. Shabana, Haifa A. S. Alhaithloul, Meaad F. Alaida, Mohamed Kh ElGhannam, Amira E. El–Sherief, Asmaa F. M. Badawy, Mohamed Shokr, Nazih Y. Rebouh, Mahmoud El-Sharkawy

**Affiliations:** 1 Soils, Water and Environment Research Institute (SWERI), Agricultural Research Center, Giza, Egypt; 2 Biology Department, Collage of Science, Jouf University, Sakaka, Saudi Arabia; 3 Agron. Dept. Sugar Crops Res. Institute. Agric. Res. Center. Giza, Egypt; 4 Soil and Water Department, Faculty of Agriculture, Tanta University, Tanta, Egypt; 5 Institute of Environmental Engineering, RUDN University, Moscow, Russia; Indian Institute of Technology (IIT) - Bombay, INDIA

## Abstract

Soil salinity remains a critical constraint to sustainable agricultural production and long-term soil resource management. A field investigation was conducted over two consecutive growing seasons (2022/2023 and 2023/2024) to evaluate the potential of glauconite-derived potassium amendments for improving soil health and enhancing sugar beet (*Beta vulgaris* L.) performance under saline soil conditions. Treatments included conventional potassium sulfate (K₂SO₄), raw glauconite powder (G), and foliar applications of glauconite extracts prepared with EDTA (GE), nitric acid (GN), and fulvic acid (GF), each applied at two concentrations (20 and 40 mL L ⁻ ¹). Among the tested treatments, GE2 and GF2 produced the most substantial improvements in soil quality, with GF2 reducing soil electrical conductivity by 35.11% and exchangeable sodium percentage by 23.33%, while increasing organic matter content and available nutrients. Soil biological indicators also responded positively, with GF2 enhancing dehydrogenase, urease, and phosphatase activities by 7.21%, 16.43%, and 48.60%, respectively. Correspondingly, GF2 achieved the highest increases in root yield (67.79%), shoot biomass (107.34%), and sugar yield (92.11%). Multivariate analyses (PCA and RDA) confirmed strong linkages between sugar yield and key soil variables, particularly available potassium, enzymatic activities, and microbial biomass. These results demonstrate that tailored glauconite-based foliar formulations—especially fulvic and EDTA extracts at higher concentrations—can serve as environmentally sound and agronomically effective measures for improving soil quality, restoring saline-affected lands, and enhancing sustainable crop productivity within the broader framework of soil and water conservation.

## 1. Introduction

Soil salinity is a significant global concern, impacting agricultural productivity and food security. According to a recent UN Food and Agriculture Organization (FAO) report, approximately 1.4 billion hectares of land worldwide are affected by soil salinity including Australia, Mexico, USA, Saudi Arabia, Egypt…etc, with an additional 1 billion hectares at risk. Salinity negatively impacts plant growth by reducing water uptake, altering nutrient availability, and causing ion toxicity, primarily through excessive sodium (Na⁺) accumulation [1]. Sugar beet exhibits moderate salt tolerance; however, excessive salinity can reduce root and shoot biomass, sugar content, and overall yield [2]. Salinity stress negatively impacts germination, root development, and leaf expansion, ultimately reducing yield and sugar content [3–5]. Egypt, being one of the leading sugar beet producers in the Middle East and North Africa, faces significant challenges due to soil salinization, particularly in reclaimed lands and coastal agricultural zones [6]. The high salt concentration in these soils adversely affects sucrose accumulation by altering carbohydrate metabolism and reducing photosynthetic efficiency [7]. Furthermore, soil salinity increases the alkalinity of the rhizosphere, leading to reduced availability of essential nutrients such as phosphorus (P), iron (Fe), and zinc (Zn), further limiting plant growth and productivity [8,9].

Sugar beet (*Beta vulgaris* L.) is one of the most important sugar-producing crops worldwide, contributing significantly to global sugar production after sugarcane crops. Worldwide, the total production of sugar beet crops reached about 271 Mt in 2020 with an increment of 131 Mt in 2008 [10]. Sugar production accounted for 80% of sugar cane, while 20% originated from sugar beet [11]. It is cultivated in temperate and semi-arid regions, where its adaptability to different climatic and soil conditions makes it a valuable crop [12]. In Egypt, sugar beet has gained increasing importance due to its higher sugar yield per unit of water consumed compared to sugarcane, making it a more sustainable option for sugar production [13]. However, soil salinity is a major abiotic stress affecting sugar beet growth and productivity, particularly in arid regions where irrigation with saline water exacerbates the problem [14].

Various soil amendments and fertilizers have been employed to mitigate the adverse effects of salinity, including organic amendments, gypsum, biochar, and mineral fertilizers [15,16]. Among these approaches, potassium (K) fertilization is critical in enhancing salinity tolerance, as it plays a crucial role in osmotic adjustment, stomatal regulation, and enzyme activation [17,18]. While potassium sulfate (K₂SO₄) is commonly used in Egyptian agriculture, alternative potassium sources such as glauconite have gained attention due to their sustainable and slow-release properties [19]. Glauconite is a green, iron-rich silicate mineral belonging to the mica group, characterized by a 2:1 layered structure with interlayer K⁺ ions weakly held within the crystal lattice [20]. Its fertilizing potential is strongly linked to its mineralogical properties, crystallo-chemical composition, and structural features, particularly the degree of exchangeable K content, interlayer charge, and Fe² ⁺ /Fe³ ⁺ ratio within octahedral sheets [21]. Glauconite typically exhibits higher K₂O contents (8–12%), greater structural stability, and a lower layer charge, which facilitates gradual potassium release through weathering processes [22]. The coexistence of Fe²⁺ and Fe³⁺ in glauconite not only governs its crystallinity and maturity but also enhances redox buffering capacity and contributes to soil biochemical activity [23]. Unlike conventional potassium fertilizers, glauconite releases K gradually, reducing nutrient leaching and enhancing long-term soil fertility. Additionally, glauconite contains major or structurally significant elements such as Fe, Mg, and Mn, which contribute to plant nutrition and soil improvement [24]. Studies have shown that glauconite application can enhance soil structure, increase cation exchange capacity (CEC), and improve water retention in sandy and saline soils, making it an eco-friendly and cost-effective soil amendment [25,26].

The originality of this study lies in the first-time use of glauconite extracts to enhance sugar beet yield and improve saline soil properties. Although glauconite has been previously investigated as a natural slow-release potassium source, its chemical modification through controlled extraction using nitric acid, EDTA, and fulvic acid has not been explored in sugar beet cultivation on salt-affected soils. Accordingly, the specific objectives of this study were to: (i) assess the effects of different glauconite extracts and application rates on soil physicochemical properties, including salinity, sodicity, nutrient availability, and hydraulic behavior; (ii) quantify their influence on soil biological activity and key enzymatic functions; (iii) evaluate the resulting responses of sugar beet growth, yield, and sugar productivity; and (iv) elucidate the relationships between soil quality indicators and crop performance using multivariate statistical analyses. Collectively, these objectives aim to provide a scientific basis for developing sustainable and cost-effective management strategies to enhance crop productivity in saline and salt-affected agroecosystems.

## 2. Materials and methods

### 2.1. Experimental layout and location

A two-year field investigation was implemented during the winter growing seasons of 2022/2023 and 2023/2024 to evaluate how various potassium sources and delivery methods influence soil characteristics and the performance of sugar beet (*Beta vulgaris* L.) in saline sodic soil. The experimental site is located at 31°25′29″ N latitude and 31°04′23″ E longitude, with an elevation of 6 meters above sea level. Field site access and research activities did not require special permits because the study was carried out on publicly accessible property and did not include interactions with regulated species or ecosystems. The soil at the location is categorized as *Vertisols*, and its baseline properties are summarized in Table 1.

**Table 1:** Mean values for physical and chemical properties of the experimental soil (0-30 cm) before cultivation in 2023 and 2024 seasons.

Traits		pH	EC (dS m^-1^)	SAR (%)	ESP (%)	A-N (mg kg^-1^)	A-P (mg kg^-1^)	A-K (mg kg^-1^)	OM (%)	Texture class	F.C (%)	W. P (%)	B.D (kg m^-3^)	PR (N cm^-2^)	IR (cm hr^-1^)	Total Mn (mg kg^-1^)	Total Fe (mg kg^-1^)	Total Zn (mg kg^-1^)	Total Cu (mg kg^-1^)
**1**^**st**^ **Season**	**Soil**	8.74^$^	4.31$	13.1	15.41	44.31	7.61	192	1.2	Clayey	42.42	21.21	1.42	388	0.7	23.11	33.24	0.28	0.5
	**Water**	7.56	0.77	3.87	–	3.74*	–	–	–	–	–	–	–	–	–	1.15	2.96	0.13	0.014
**2**^**nd**^ **Season**	**Soil**	8.65^$^	4.25$	12.22	14.51	44.46	8.82	225	1.25	Clayey	44.44	22.22	1.4	369	0.72	22.25	30.58	0.23	0.52
	**Water**	7.44	0.65	3.88	–	3.77*	–	–	–	–	–	–	–	–	–	1.39	3.17	0.12	0.016

*: total nitrogen, ^**$**^: soil paste extraction, **EC**: electrical conductivity, **SAR**: sodium adsorption ratio, **ESP**: exchangeable sodium percentage, **OM**: organic matter, **PR**: penetration resistance, **W.P**: water welting point, **F.C**: field capacity, **IR**: hydraulic conductivity.

The trial layout consisted of 36 individual plots, each measuring 15 meters in length and 5 meters in width, arranged in a completely randomized design with four replications. Potassium treatments were introduced either via soil incorporation—using potassium sulfate or glauconite powder—or by foliar spraying of glauconite extracts derived using different chemical agents. The applied treatments included

1. Control without potassium addition (CK).
2. Potassium sulfate applied at the recommended rate of 24 kg K fed ⁻ ¹ (K).
3. Glauconite powder applied at 100% of the recommended potassium rate (G).
4. Foliar application of 20 ml L ⁻ ¹ glauconite extracted with nitric acid (GN1).
5. Foliar application of 40 ml L ⁻ ¹ glauconite extracted with nitric acid (GN2).
6. Foliar application of 20 ml L ⁻ ¹ glauconite extracted with EDTA (GE1).
7. Foliar application of 40 ml L ⁻ ¹ glauconite extracted with EDTA (GE2).
8. Foliar application of 20 ml L ⁻ ¹ glauconite extracted with fulvic acid (GF1).
9. Foliar application of 40 ml L ⁻ ¹ glauconite extracted with fulvic acid (GF2).

All materials used in the experimental treatments were of analytical grade to ensure consistency and reproducibility. Potassium sulfate (K₂SO₄; 48% K₂O, ≥ 65% purity, Sigma-Aldrich), nitric acid (95% purity, Sigma-Aldrich), and Ethylenediaminetetraacetic acid (EDTA, ≥ 99% purity, Sigma-Aldrich) applied in treatment with no detectable heavy-metal contamination, while Fulvic acid (≥99.9% purity) was derived from a Microbiology Department; Soils, Water and Environment Research Institute, Agricultural Research Center, Sakha Agriculture Research Station, Kafr El-Sheikh, Egypt.

Prior to planting, the soil was prepared by mechanical plowing and deep subsoiling (45 cm depth, 1.5 m apart). Precision laser leveling was performed to achieve a 0.1% field gradient, ensuring uniform irrigation across 60-meter furrows. Seeds of sugar beet cultivar “pleno” were sown during the first week of October in both seasons at a seeding rate of 4 kg fed ⁻ ¹. Plants were thinned at the four-leaf stage to one per hill before the initial irrigation.

**Fig 1:**
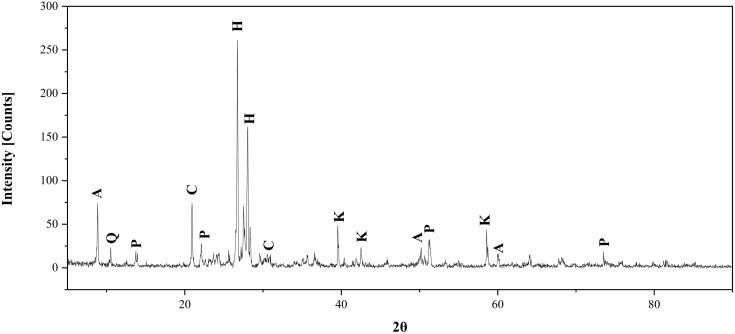
X-Ray diffraction of Glauconite used in the experiment, denoted as K: Potash (K_2_CO_3_), Q: Quartz (SiO_2_), A: Aluminum-Iron (AlFe_2_), C: calcite (CaCO_3_), and P: polyhalite (K_2_Ca_2_Mg(SO_4_)_4_).

Basal fertilization included the application of phosphorus as calcium superphosphate (15.5% P₂O₅) at 200 kg fed ⁻ ¹ during land preparation. Nitrogen, in the form of urea (46% N), was split into two doses totaling 90 kg N fed ⁻ ¹—one following thinning and the other applied a month later. Potassium sulfate (48% K₂O) was used only in designated K-treated plots at a rate of 50 kg fed ⁻ ¹, delivered with the first irrigation. Glauconite powder, with a K₂O content of 5.40%, was air-dried, ground, and applied at a rate of 480 kg fed ⁻ ¹ to match the potassium supplied by K₂SO₄. The chemical composition of raw glauconite used in this study was thoroughly characterized prior to its application showing its total contents of: Fe₂O₃ (15.17%), CaO (4.23%), MnO (0.05%), Al₂O₃ (1.04%), SiO₂ (52.26%), TiO₂ (0.51%), MgO (1.17%), P₂O₅ (0.24%), Na₂O (0.75%), and loss on ignition (9.18%). Mineralogical identification was performed using X-ray diffraction (XRD), and the resulting diffractogram illustrated in (Fig 1) confirmed the heterogeneous mineral assemblage of the glauconite material. The dominant crystalline phases included glauconitic mica associated with quartz (SiO₂) as the primary silicate phase, along with calcite (CaCO₃), hematite (Fe₂O₃), aluminum–iron mixed oxides, potash-bearing phases (K₂CO₃), and polyhalite (K_2_Ca_2_Mg(SO_4_)_4_). This mineralogical composition supports the role of glauconite as a multi-nutrient, potassium-bearing material with potential contributions of Ca, Mg, Fe, and S to soil fertility.

Glauconite extraction was carried out under controlled laboratory conditions to ensure reproducibility and consistency among treatments. A fixed solid-to-liquid ratio of 1:5 (w/v) was maintained by suspending 200 g of finely ground glauconite in 1.0 L of extraction solution. Three extractants were employed. Nitric acid extraction (GN) was performed using 0.5 M HNO₃ (pH ≈ 0.3), representing a strong inorganic acid capable of promoting proton-driven dissolution of K-bearing mineral phases. Chelating extraction (GE) employed a mixed solution consisting of 0.5 M EDTA (pH adjusted to ~4.5), 0.5 M acetic acid (pH ≈ 2.9), and 0.5 M ammonium acetate (pH ≈ 4.7), intended to mobilize exchangeable and weakly bound cations through chelation and ion-exchange processes while limiting excessive destruction of the glauconite lattice. Fulvic acid extraction (GF) was conducted using high-purity fulvic acid (99.9%) dissolved in deionized water at its natural acidic pH (≈3.5–4.0), simulates organic ligand–mediated weathering and facilitating selective nutrient release. Both EDTA and Fulvic acid were employed solely as extractants to enhance the dissolution and mobilization of K and associated micronutrients from glauconite and were not intended as independent bioactive inputs. Each suspension was placed in sealed 1 L flasks and agitated on a mechanical shaker at 4000 rpm for 24 h at room temperature (25 ± 2 °C) to allow sufficient dissolution and mobilization of potassium and associated nutrients from the glauconite matrix. Upon completion of extraction, the suspensions were allowed to settle briefly and then filtered through Whatman No. 42 filter paper to remove residual solids. The obtained clear filtrates were immediately transferred into dark, airtight bottles and stored at 4 °C until foliar application to prevent chemical alteration. The chemical composition of the resulting glauconite extracts is presented in Table 2.

**Table 2:** Extractable Glauconite properties.

Treatments	pH	EC	N	P	K	Na	Cu	Fe	Mn	Zn
	–	dS m^-1^	%	%	Meq L^-1^	Meq L^-1^	ppm	ppm	ppm	ppm
**GN**	1.60	28.30	2.61	0.013	22.6	218.00	0.25	3983.00	271.30	3.26
**GE**	4.73	21.20	1.80	0.004	18.6	260.00	0.48	1824.50	934.20	9.65
**GF**	1.13	22.30	2.60	0.022	25.1	384.00	12.14	4302.00	499.50	44.37

GE: Glauconite extracted with nitric acid, **GN**: Glauconite extracted with EDTA, **GF**: Glauconite extracted with fulvic acid.

Harvesting took place 180 days after sowing, in late April of each respective season. All crop management practices followed standard agronomic recommendations issued by Egypt’s Ministry of Agriculture for sugar beet cultivation in the Nile Delta.

### 2.2. Soil characteristics

After crop harvest, composite soil samples were obtained from the surface layer (0–30 cm) of each treatment plot. The collected samples were air-dried under shade, ground to a fine consistency, and passed through a 2 mm sieve to standardize particle size before analysis. Soil reaction (pH) was determined using a 1:2.5 soil-to-water suspension with a calibrated digital pH meter (Model H12211-02, Thermofisher, HANNA, Waltham, MA, USA). Electrical conductivity (EC) was measured in a 1:5 soil-to-water ratio using a conductivity meter (Model CON2700, EUTECH, Illinois, USA), following the protocol described by [27].The determination of organic matter (OM) content was performed via the wet oxidation technique using potassium dichromate (K₂Cr₂O₇), as outlined by [28].Soil cation exchange capacity (CEC) was measured using ammonium acetate extraction, adopting the method of [29]. Available macronutrients—including phosphorus (P) and potassium (K)—were analyzed using established procedures [30], while available forms of nitrogen (ammonium and nitrate) were determined through the Kjeldahl digestion approach, in accordance with [31]. Bulk density (BD) and total porosity (TP) were calculated following the methods developed by Campbell et al. (2000). The resistance of the soil to penetration (SPR) was measured in situ with a handheld penetrometer following the standardized protocol of [32]. The saturated hydraulic conductivity (Ks) was determined using the constant head method as recommended by [33]. Calcium carbonate (CaCO₃) content was assessed volumetrically with a calcimeter following [34]. To evaluate total concentrations of heavy metals—iron (Fe), manganese (Mn), copper (Cu), and zinc (Zn)—soil samples underwent acid digestion using a mixture of concentrated H₂SO₄ and H₂O₂. The digested extracts were subsequently analyzed using inductively coupled plasma (ICP) spectroscopy (ICP-ISO Prodigy Plus), according to the guidelines provided by [30]. Soil sodicity was assessed through the calculation of the exchangeable sodium percentage (ESP), following the procedure described by [35].

### 2.3. Measurement of plant productivity and quality attributes

At physiological maturity, ten representative plants were randomly chosen from each plot to assess biomass and chemical composition. Quantitative productivity indicators such as shoot and root yields (t fed ⁻ ¹) were determined using the protocol reported by [36]. Sugar yield (kg fed ⁻ ¹) was calculated by multiplying root biomass by the corresponding sugar percentage, in line with [37].The concentration of sugar in freshly extracted root juice was quantified using a portable refractometer (Model BK-PR, Biobase, Shandong, China), adhering to the procedure outlined by [38]. To estimate sugar losses to molasses (SLM%), the approach of [39] was applied based on the concentrations of key root constituents.

### 2.4. Statistical analysis

All collected data were subjected to statistical evaluation through a two-factor analysis of variance (ANOVA), incorporating four replications per treatment. The analysis was conducted using IBM-SPSS Statistics software (Version 29). Replication effects were considered random, while treatments and other factors were modeled as fixed. A significance level of p < 0.05 was used to detect differences among means, and post hoc comparisons were performed using Duncan’s Multiple Range Test (DMRT).

To further interpret interrelationships within the dataset, advanced multivariate tools were employed. Principal Component Analysis (PCA), Redundancy Analysis (RDA), and Spearman’s rank correlation were conducted using MATLAB [Version R2022a], enabling visualization of the associations among soil, plant, and productivity variables.

## 3. Results

### 3.1. Effect of glauconite amendments on soil properties

The data in Table 3 shows the effect of glauconite, potassium sulfate, and different types of glauconite extracts on soil physicochemical properties. The ANOVA analysis manifested significant (p < 0.01) effects of different treatments on soil properties except for bulk density in both seasons. Both EC and ESP explored the significant effects of treatments, seasons, and their combinations. The application of GF2 recorded the lowest values of pH in both seasons. The addition of GE2 recorded the best treatment in enhancing soil EC, ESP, and Ks in both seasons recording reduction percentages of 35.11% and 23.33% for EC and ESP, and an increment of 56.55% in Ks in the average of the two seasons compared to control. On the other hand, the application of glauconite (G) revealed the enhancements in soil OM recording values of 2.36% and 2.42% for the first and second seasons respectively with an average increment of 104.31% compared to the control. In comparison, it recorded the lowest values of SPR with values of 250 and 240 N cm^-2^ for the first and second seasons respectively with an average reduction of 34.67% compared to the control, while GF application recorded the highest values of hydraulic conductivity (Ks) compared to other treatments with GF2 registered the best record of 1.16 cm h^-1^.

**Table 3:** Effect of different K- sources and glauconite extracts on soil physicochemical properties after two successful seasons of sugar beet plants.

Treatments	Season 1							Season 2						
	pH	EC	OM	ESP	BD	Ks	SPR	pH	EC	OM	ESP	BD	Ks	PR
CK	8.64^a^	4.76^a^	1.16^d^	13.44^a^	1.45^a^	0.72^c^	380.00^a^	8.64^a^	4.74^a^	1.18^c^	12.10^a^	1.44^a^	0.73c	370.00^a^
K	8.54 cd	3.75^c^	2.03^b^	11.68^b^	1.39^a^	0.79^bc^	280.00^bcde^	8.52^d^	3.75^c^	2.04^b^	10.80^c^	1.38^a^	0.81bc	270.00^bcd^
G	8.53^d^	3.36^h^	2.36^a^	10.57b^c^	1.34^a^	1.08a	250.00^e^	8.52^d^	3.35^h^	2.42^a^	10.21^h^	1.33^a^	1.11a	240.00^d^
GE1	8.59b	3.79b	1.45 cd	10.87bc	1.43a	0.91bc	350.00a	8.58b	3.78b	1.47c	10.85b	1.42a	0.92bc	340.00a
GN1	8.55 cd	3.74d	1.47 cd	10.78bc	1.43a	0.88bc	350.00a	8.54 cd	3.72d	1.50c	10.76d	1.43a	0.91bc	350.00a
GF1	8.44e	3.60e	1.85b	10.59bc	1.42a	0.92b	340.00abc	8.43e	3.59e	1.93b	10.57e	1.41a	0.94b	340.00a
GE2	8.56c	3.08i	1.48 cd	9.79c	1.41a	1.13a	330.00abcd	8.55c	3.08i	1.50c	9.80i	1.41a	1.14a	330.00ab
GN2	8.53d	3.53f	1.50c	10.48bc	1.42a	1.12a	340.00abc	8.52d	3.51f	1.51c	10.45f	1.41a	1.13a	330.00ab
GF2	8.33f	3.48g	1.94b	10.41c	1.41a	1.16a	340.00ab	8.32f	3.45g	1.96b	10.36g	1.40a	1.16a	330.00ab
LSD (0.05)	1.01	0.46	0.20	0.76	0.17	0.11	39.77	1.01	0.46	0.27	0.01	0.17	0.11	38.87
ANOVA	**pH**		**EC**		**OM**		**ESP**		**BD**		**Ks**		**SPR**	
Treatment	**		**		**		**		NS		**		**	
Season	**		**		NS		**		NS		NS		NS	
Season × Treatment	NS		**		NS		**		NS		NS		NS	

**EC:** Electrical conductivity (dS m^-1^), **ESP:** Exchangeable sodium percentage (%), **CEC:** Cations exchange capacity (Cmol kg^-1^), **OM**: organic matter (%), **B.D:** Bulk density (g cm^-3^), **SPR**: Soil penetration resistance (N cm^-2^), IR: infiltration rate (cm h^-1^). means in the same columns with different letter are significantly different at (0.05) level. ** Significant difference at 0.01 probability levels, **NS**: not significant.

As for soil nutrient conditions, Table 4 illustrates that different treatments affected significantly (p < 0.01) in all measured nutrients except for Mn element. ANOVA analysis showed high effects of treatments, seasons, and their interactions with both NO_3_ and Fe availability in soil. The nitrogen content comprised NO_3_ and NH_4_ changed with different treatments and seasons and registered the highest values with GE2 and G treatments for NH_4_ and NO_3_ in the first season recording 58.48 and 43.22 mg Kg^-1^ respectively, while G and GF1 treatments registered the highest values of NH_4_ and NO_3_ in the second season recording 61.92 and 51.54 mg Kg^-1^ respectively. The application of G explored great effects in enhancing total N content in both seasons recording an average increment of 80.94% compared to control. The usage of GF2 explored a maximum effect on boosting K availability in soil with an average enhancement of 132.90% compared to control. As for micronutrient availability in soil, the GE2 treatment recorded the highest values of Fe, Mn, Zn, and Cu contents in both seasons with average increments of 1.5, 3.3, 0.9, and 3.0 times respectively compared to the control followed by GE1.

**Table 4:** Effect of different K- sources and glauconite extracts on soil nutrient availability (mg kg^-1^) after two successful seasons of sugar beet plants.

Treatments	Season 1							Season 2						
	NH_4_	NO_3_	K	Fe	Mn	Zn	Cu	NH_4_	NO_3_	K	Fe	Mn	Zn	Cu
CK	27.60^f^	20.67^d^	181.41^h^	3.96^h^	23.01^i^	0.176i	0.19^g^	34.45^d^	17.23^e^	262.81^g^	3.75^h^	23.13^i^	0.18^i^	0.19^i^
K	32.45^e^	28.790^c^	398.44^b^	4.66dg	35.34^h^	0.184^h^	0.27^f^	34.43^d^	34.43^b^	506.78d	4.56^g^	35.34^h^	0.19^h^	0.27^h^
G	44. 76 ^c^	43.22^a^	374. 74 e	5.50^f^	58.98^g^	0.22^c^	0.34^e^	61.92^a^	30.96^c^	470.34^f^	5.47^f^	59.03^g^	0.36^c^	0.47^g^
GE1	34.45d	34.44b	371.19f f	9.35b	93.05b	0.26b	0.66b	34.44d	34.44b	495.06e	9.21b	93.15b	0.41b	0.81b
GN1	34.44d	34.50b	353.41g	6.90c	62.67f	0.20g	0.66b	51.76c	17.25e	470.35f	6.79c	62.66f	0.30g	0.76d
GF1	34.43d	34.44b	395.15c	5.83e	71.96d	0.21e	0.62c	34.44d	51.54a	506.50d	5.81e	72.16d	0.31e	0.72f
GE2	58.48a	17.22e	388.74d	9.51a	98.59a	0.26a	0.67a	58.86b	17.22e	518.47c	9.44a	98.71a	0.42a	0.82a
GN2	34.44d	34.50b	399.13b	6.89c	63.23e	0.207f	0.67a	51.76c	17.35d	531.19b	6.77c	63.22e	0.31f	0.77c
GF2	51.61b	34.44b	455.59a	6.46 d	73.14c	0.215d	0.62b	51.66c	34.42b	579.02a	6.40d	73.36c	0.34d	0.74 e
LSD (0.05)	0.08	0.01	1.08	0.02	0.18	0.001	0.001	0.15	0.006	0.82	0.01	0.09	0.001	0.002
ANOVA	**NH**_**4**_		**NO**_**3**_		**K**		**Fe**		**Mn**		**Zn**		**Cu**	
Treatment	**		**		**		**		NS		**		**	
Season	**		**		NS		**		NS		NS		NS	
Season × Treatment	NS		**		NS		**		NS		NS		NS	

means in the same columns with different letter are significantly different at (0.05) level. ** Significant difference at 0.01 probability levels, **NS**: not significant.

**Fig 2:**
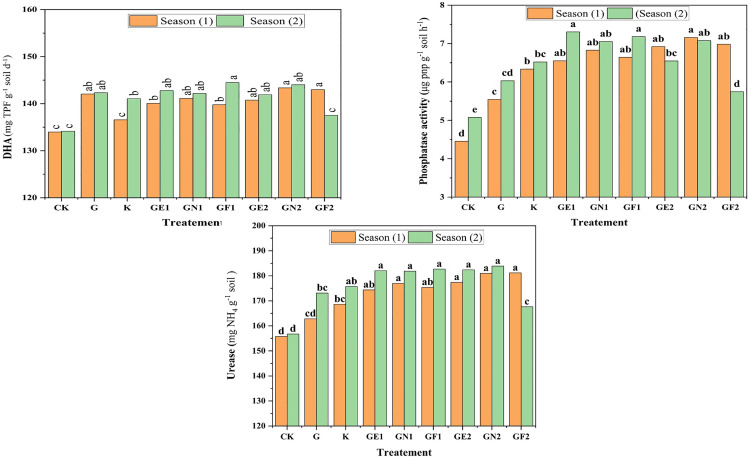
Effect of different K-sources and glauconite extracts in soil dehydrogenase (DHA), urease, and phosphatase enzyme activities after two successful seasons of sugar beet plants.

As for soil enzymatic effects, the application of glauconite extracted by fulvic acid (GF) resulted in the ameliorating of DHA enzymes in both seasons as appeared in (Fig 2). The high doses of different extracts especially GN and GE were recorded as having the best impacts in enhancing soil phosphatase and urease activities with GN2 registering the best results recording compared to the control treatment improvements of 49.33% for phosphatase, and 16.77% for urease enzyme in the average of two seasons respectively.

**Fig 3:**
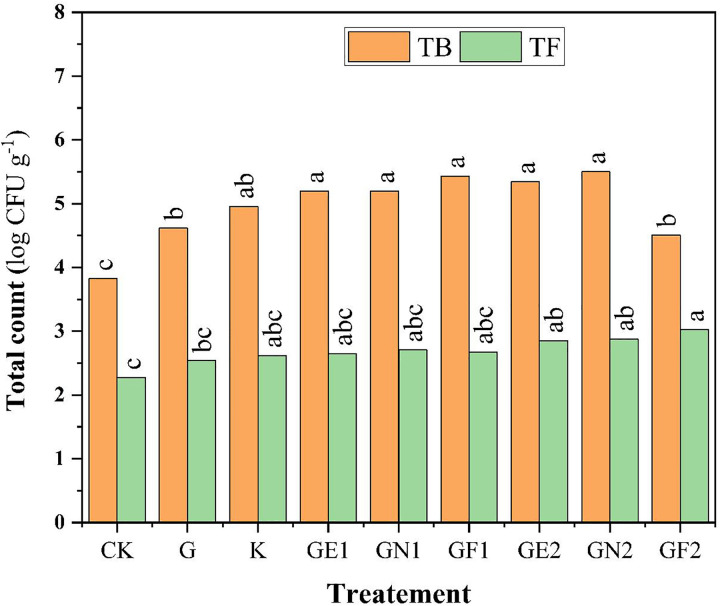
Total counts of bacteria (TB) and total count of fungi (TF) as affected by different K-sources and glauconite extracts after two successful seasons of sugar beet plants.

The microbiological activities comprising the total count of bacteria (TB) and the total count of fungi (TF) as responses for different treatments in the average of the two seasons are investigated in (Fig 3). The data illustrated all glauconite extracts enhanced the total count of microbes in soil with no significant differences compared to powder glauconite (G) and potassium sulfate (K). The chart indicated that GN2 has superior effects in increasing the total count of bacteria, while GF2 registered the maximum count of fungi on average in both seasons.

### 3.2. Effect of glauconite amendments on sugar beet plants

The data in Table 5 indicates that both treatments and seasons affected significantly (p < 0.01) sugar beet yield traits. The data illustrated that the application of GF2 listed the highest treatment in ameliorating sugar beet root yield, shoot yield, and sugar yield in both seasons recording average enhancements of 67.79%, 107.33%, and 92.11% compared to control in both seasons followed by K treatment. The extracted sugar contents increased by 31.45% with the application of G treatment in the first season and changed in the second season to register the highest record with K treatment with 11.42% compared to the control. The sugar lost to molasses (SLM) explored an increase with GF2 treatment recording 2.73% and 2.65% for first and second seasons respectively. It has been noticed that the root yield and sugar yield improved in the second season compared with the first season with best enhancement explored with the application of GF2 treatment recorded increase of 1.63 and 0.83 t fed^-1^ respectively.

**Table 5:** Sugar beet yield and productivity parameters as affected by K- sources and glauconite extracts after two successful seasons.

Treatments	1^st^ Season					2^nd^ Season				
	Root yield (t fed ^−1^)	Shoot yield (t fed ^−1^)	Sugar yield (t fed ^−1^)	Extracted sugar (%)	SLM (%)	Root yield (t fed ^−1^)	Shoot yield (t fed ^−1^)	Sugar yield (t fed ^−1^)	Extracted sugar (%)	SLM (%)
**CK**	15.64h	4.30d	2.26h	11.42h	2.43h	16.98e	5.44c	2.98i	14.32f	2.20g
**K**	23.80b	6.38b	4.03b	13.81c	2.52g	25.27b	8.05b	4.90b	15.96a	2.47f
**G**	18.44d	4.94d	3.32d	15.01a	2.42i	19.57d	6.24c	3.78d	15.78b	2.59c
**GE1**	16.51g	4.42g	2.73g	13.35f	2.59e	17.52e	5.58c	3.08h	14.10g	2.54d
**GN1**	16.50g	4.43g	2.74g	13.41e	2.57f	22.33c	5.59c	3.30g	15.37d	2.52e
**GF1**	21.04c	5.64c	3.60c	13.82c	2.69b	17.51e	7.12bc	4.30c	15.66c	2.64a
**GE2**	17.63e	4.72e	2.91f	13.29g	2.61c	18.71de	5.96c	3.43f	14.80e	2.58c
**GN2**	17.47f	4.69f	2.94e	13.61d	2.60d	18.54de	5.92c	3.51e	15.39d	2.60b
**GF2**	26.55a	10.40a	4.61a	14.02b	2.73a	28.18a	9.81a	5.44a	15.70c	2.65a
**LSD (0.05)**	0.004	0.004	0.42	0.006	0.006	1.21	1.21	0.01	0.04	0.007
**ANOVA**	**Root yield (t fed** ^**−1**^)		**Shoot yield (t fed** ^**−1**^)		**Sugar yield (t fed** ^**−1**^)		**Extracted sugar (%)**		**SLM (%)**	
**Treatment**	**		**		**		**		**	
**Season**	**		**		**		**		**	
**Season × Treatment**	*		NS		**		**		**	

**SLM**: sugar losses to molasses, means in the same columns with different letter are significantly different at (0.05) level. **, * Significant difference at 0.01 and 0.05 probability levels. **NS**: not significant.

## 4. Discussion

### 4.1. Soil properties

The results demonstrated that the application of glauconite, potassium sulfate, and different glauconite extracts significantly influenced soil physicochemical properties, with the exception of bulk density, which remained unaffected across both seasons as appeared in Table 3. These findings highlight the potential of glauconite-based treatments in improving saline soil conditions, which is crucial for enhancing crop productivity in salt-affected soils. The application of GF2 consistently recorded the lowest soil pH values across both seasons. This effect could be attributed to the chelating properties of fulvic acid, which enhances nutrient solubility and mobility, thus lowering soil pH [40]. The decrease in pH with GF2 suggests improved nutrient availability and root uptake efficiency, promoting better crop growth under saline conditions. The significant reduction in electrical conductivity (EC) and exchangeable sodium percentage (ESP) observed with GE2 suggests that EDTA-extracted glauconite effectively reduced soil salinity and sodicity. Specifically, GE2 decreased EC by 35.11% and ESP by 23.33% compared to the control, which aligns with previous studies reporting the role of chelating agents in enhancing ion exchange and leaching of excess salts [41,42]. The improved soil desalination can be linked to the increased solubility of potassium from glauconite, enhancing ionic balance and reducing sodium accumulation [43].Additionally, the enhancement occurred in hydraulic conductivity (Ks) with GE2 application, indicates enhanced soil structure and permeability. This enhancement could be due to the improvement in soil aggregation and pore connectivity facilitated by EDTA action of chelating, which promotes the leaching of excess sodium and improves soil aeration [44].The superior performance of GE2 in enhancing soil EC, ESP, and Ks highlights the effectiveness of EDTA as an extraction agent, possibly due to its strong chelating capacity, which enhances potassium availability and sodium displacement. In contrast, GF2 showed greater efficacy in reducing soil pH, which could be linked to the acidic nature of fulvic acid, promoting the release of hydrogen ions as confirmed by the acidic nature in extract shown in Table 2 with value of 1.13. On the other hand, the application of glauconite powder (G) significantly increased soil organic matter (OM) content in both seasons, with around two times compared to the control. This result could be attributed to the ability of glauconite to improve soil microbial activity and organic matter decomposition, contributing to enhanced soil fertility [25]. Moreover, G recorded the lowest soil penetration resistance (SPR), with an average reduction around 1.5 times compared to the control. The decrease in SPR indicates improved soil permeability and reduced compaction, which are essential for better root penetration and growth. The improved soil structure may be due to the enhanced organic matter content and potassium availability, which promote better soil aggregation and stability [45]. This suggests that solid glauconite is more effective in improving soil structure, whereas its extracts (especially GE2 and GF2) are more effective in ameliorating soil salinity and sodicity. Both treatments and seasons influenced the soil nutrient availability which is critical for sustaining crop productivity. The increased NH₄ ⁺ availability observed with the application of EDTA-extracted glauconite (GE2) and solid glauconite (G) could be attributed to improved cation exchange capacity and reduced nitrogen leaching due to better soil aggregation and structure [46,47]. For NO₃ ⁻ , the highest concentrations were recorded with GE2 in the first season and GF1 in the second season. This variation might be due to either the high nitrogen content in GE and GF as appeared in Table 2 recording 1.80% and 2.60% respectively, or their effects on nitrogen mineralization and microbial activity, which influence the nitrification process. The chelating agents in GE2 and GF1 possibly enhanced the solubilization and availability of nitrogen compounds, leading to higher NO₃ ⁻ concentrations [48]. Generally, the application of solid glauconite (G) significantly improved total nitrogen content, showing an average increase of 1.8 times than the control treatment. This improvement is likely due to slow-release mechanism of glauconite, which maintains a steady supply of potassium and other nutrients that support microbial activity and nitrogen cycling [20]. Despite that, the GF2 exhibited the most substantial impact on soil potassium availability, which appear the ability of fulvic acid as a natural chelating agent, increasing nutrient uptake by plants and minimizing potassium fixation in clay minerals [49] as well as the high interior content of K in GF with value of 25.10 Meq L^-1^. This enhanced potassium availability is particularly beneficial under saline conditions, where sodium ions compete with potassium for uptake by plant roots. As for micronutrient availability, the GE2 in Table 2 consistently recorded the highest concentrations of Fe, Mn, Zn, and Cu across both seasons, which could be attributed to the prevention effect of EDTA to micronutrient precipitation and promotion of their mobility in the soil solution particularly under saline conditions where micronutrient availability is often limited due to high pH and ionic competition [50]. This process suggests improved redox conditions and micronutrient solubility, which are crucial for various physiological processes in plants, including photosynthesis and enzyme activation. These findings underscore the potential of using glauconite extracts, particularly EDTA and fulvic acid, as effective soil amendments for improving nutrient availability under saline conditions.

The application of glauconite extracts significantly influenced soil enzymatic activities and microbial populations. High doses of fulvic acid (GF), nitric acid (GN), and EDTA (GE) showed the most pronounced effects on soil DHA, phosphatase, and urease activities which is indicative of improved soil biochemical functioning. Dehydrogenase activity (DHA), a key indicator of microbial oxidative metabolism, was significantly increased by glauconite extracted with fulvic acid (GF), which could be attributed to its role in enhancing soil physical properties by decreasing soil SPR, BD, and increasing Ks, resulting the enhancing of soil aggregation, respiratory, and microbial energy, subsequently boosts DHA levels [51]. Additionally, fulvic acid improves nutrient availability and promotes a favorable environment for enzyme-producing microbes [52].The ability of nitric acid to release bound nutrients, resulted in increasing substrate availability, yielded the capability of GN2 in booting soil phosphatase and urease activities. This resulted in increasing the bacterial population of soil with GN2 application, which is linked to the role of nitric acid in increasing nitrate availability, which serves as an essential nitrogen source for bacteria [53]. Furthermore, chelating properties in GE extracts enhance micronutrient solubility, supporting microbial growth and enzymatic synthesis [54,55]. The enhanced phosphatase activity suggests improved phosphorus mineralization, while increased urease activity indicates enhanced nitrogen cycling and availability [56]. Moreover, the increased fungal population observed with GF2 suggests that fulvic acid positively influences fungal growth by enhancing organic matter decomposition and nutrient cycling [57]. The differential effects observed between bacterial and fungal populations suggest that these extracts selectively influence microbial community dynamics, potentially leading to changes in soil nutrient cycling and plant nutrient availability. The increased enzymatic activities and microbial populations highlight the potential of glauconite extracts to improve soil health and fertility, especially under saline soil conditions.

### 4.2. Sugar beet productivity

The significant improvement in sugar beet (*Beta vulgaris* L.) yields components with GF2 treatment employed increments of 1.7 times for root yield, 2.1 times for shoot yield, and 1.9 times for sugar yield compared to the control across both seasons, followed by potassium sulfate (K). Fulvic acid is known to enhance nutrient uptake efficiency, improve root development, and stimulate plant growth hormones [58]. The superior performance of GF2 in boosting root, shoot, and sugar yield suggests that the higher dose of fulvic acid extract provided a more favorable nutrient environment, leading to enhanced photosynthetic activity and biomass accumulation [59]. Potassium sulfate (K) also showed notable yield improvements, likely due to essential role of potassium in osmoregulation, enzyme activation, and carbohydrate translocation, which are crucial for sugar beet growth and sugar accumulation [18]. The significant increase in extracted sugar content observed with G in the first season and with K in the second season further supports the importance of adequate potassium supply in enhancing sugar synthesis and storage in beet roots. The observed seasonal variation in yield performance, with better results in the second season, could be attributed to the residual effects of those treatments which influence crop growth dynamics and nutrient uptake efficiency [60]. The GF2 treatment consistently showed the highest yield enhancements across both seasons, indicating its effectiveness in stabilizing yield performance under varying seasonal conditions. The increase in sugar losses to molasses (SLM) with GF2 may be linked to the higher potassium content associated with fulvic acid extracts, as increased potassium levels can lead to higher SLM due to its impact on sugar crystallization [61]. However, the overall yield gains observed with GF2 outweigh the slight increase in SLM, highlighting its potential as an effective soil amendment for enhancing sugar beet productivity. This suggests that fulvic acid-extracted glauconite can be a valuable alternative to conventional potassium fertilizers, offering additional benefits of improved nutrient availability and soil health enhancement.

### 4.3. The dynamic performance of glauconite extracts

**Fig 4:**
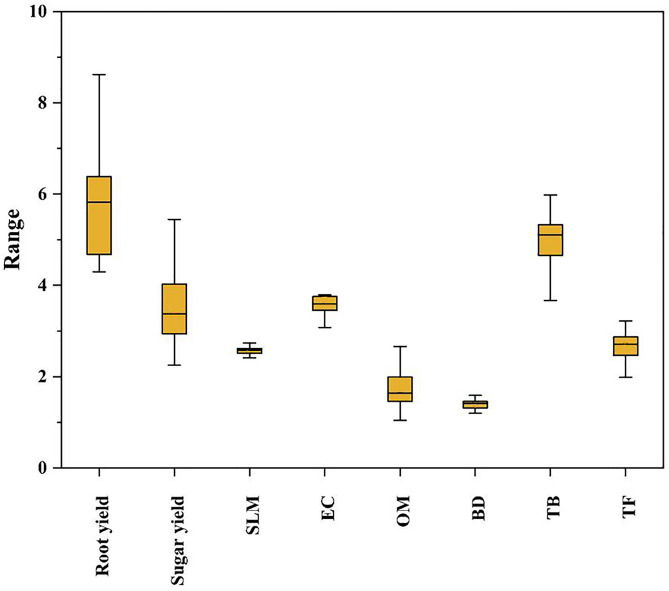
Box-plot analysis of root yield (t fed ^−1^), sugar yield (t fed ^−1^), SLM (%), electrical conductivity (EC, dS m^-1^), organic matter (OM, %), bulk density (BD, g cm^-3^), total count of bacteria (TB, CFU g^-1^), and total count of fungi (TF, CFU g^-1^).

The boxplot in (Fig 4) illustrates the variation in sugar beet yield components (root yield, sugar yield, SLM), soil physicochemical properties (EC, OM, BD), and microbial activity indicators (TB, TF) under different treatments. The results revealed that the root yield exhibited the widest range, indicating a high variability among treatments, with the median directed towards the upper range, suggesting that treatments notably enhanced root yield, possibly due to improved nutrient availability and enhanced soil structure [62]. while the sugar yield showed a narrower distribution compared to root yield but still displayed significant variability, reflecting the impact of treatments on sugar accumulation. On the other hand, SLM displayed the least variability, indicating consistent effects of treatments on SLM, which could be linked to the balanced potassium availability influencing sugar crystallization [63].As for soil biochemical impacts, the EC showed a relatively narrow range, suggesting that treatments moderately affected soil salinity. Treatments that included fulvic and EDTA extracts likely improved salt leaching through enhanced soil structure and increased infiltration rates [64].While OM exhibited a wider range, highlighting the differential influence of treatments on soil organic carbon content, suggesting that treatments such as glauconite powder and fulvic acid extracts contributed to organic matter accumulation, enhancing soil fertility and microbial activity [65], and BD showed minimal variability, suggesting that treatments had limited impact on soil compaction likely due to the short-term application period. However, the observed changes in organic matter may lead to long-term improvements in soil structure and reduced bulk density [66]. Total fungi (TF) showed a narrower range compared to TB, suggesting a more consistent effect on fungal populations across treatments. The high variability in TB suggests that glauconite extracts differentially influenced bacterial populations, possibly due to variations in nutrient availability and pH changes induced by the treatments [67].The narrower range in TF indicates a more consistent effect on fungal populations, which might be attributed to the stable organic matter inputs enhancing fungal growth [68]. The overall increase in microbial activity, as indicated by TB and TF, can be linked to enhanced nutrient cycling and soil health, promoting better plant growth and yield [69].

**Fig 5:**
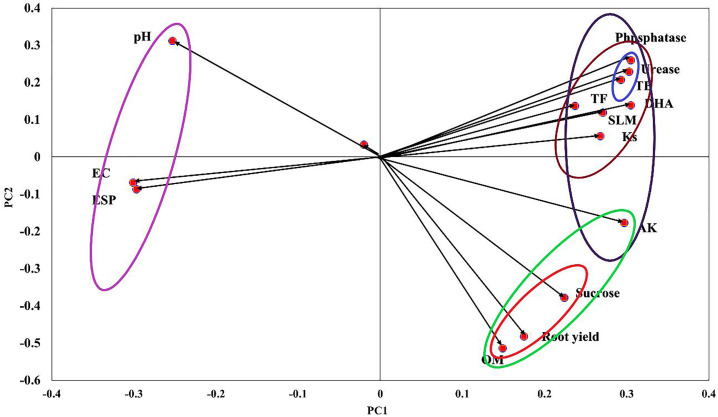
Principal component analysis (PCA) of different soil properties and sugar beet plant properties as affected by different K-sources and glauconite extracts after two successful seasons of sugar beet plants.

The Principal Component Analysis (PCA) biplot in (Fig 5) illustrates the relationships between soil enzymatic activities (DHA, Urease, Phosphatase), microbial populations (Total Bacteria (TB), Total Fungi (TF)), soil traits (pH, EC, ESP, OM, Ks, AK), and sugar beet yield components (Shoot yield, Root yield, Sugar yield, SLM). The first principal component (PC1) explains 55.30% of the variance, while the second component (PC2) accounts for 12.20%, collectively explaining 67.50% of the total variance. The data revealed the high correlation between root and sugar yield with soil available K and organic matter content, with negative impact occurred with soil pH, EC, and ESP. The positive association suggests that higher levels of AK and OM significantly enhance sugar beet productivity. Available potassium plays a crucial role in sugar translocation and water regulation in plants, which enhances sugar yield and root development [70]. Organic matter improves soil structure, water-holding capacity, and nutrient availability, fostering better root growth and higher yield [71]. High soil pH, salinity (EC), and sodicity (ESP) negatively affect nutrient availability, leading to reduced plant growth and yield. Elevated pH levels decrease the solubility of micronutrients such as iron, manganese, and zinc, which are essential for sugar beet growth [72]. High EC levels result in osmotic stress, reducing water uptake and impairing photosynthesis, ultimately leading to lower yield [73]. The negative impact of ESP is related to poor soil structure and reduced hydraulic conductivity (Ks), which hinder root penetration and nutrient absorption [74]. These findings align with earlier research that reported yield reduction in sugar beet under saline-sodic soil conditions due to limited nutrient uptake and osmotic stress [19]. The chart also indicates strong positive correlation among soil enzymes (DHA, Urease, and phosphatase) and soil microbial activity (TB, and TF) and both soil hydraulic conductivity (KS) and SLM. Soil enzymes such as DHA, Urease, and Phosphatase are key indicators of microbial activity and soil health. These enzymes facilitate nutrient cycling, including carbon, nitrogen, and phosphorus, which are essential for plant growth [75]. The observed positive correlation suggests that increased microbial activity enhances soil enzymatic functions, which in turn improves soil hydraulic conductivity (Ks). This improvement is likely due to the enhanced soil structure and aggregate stability facilitated by microbial exudates and enzyme actions, leading to better water infiltration and movement through the soil profile [47]. The positive association with SLM indicates that improved soil biological activity and hydraulic properties positively influence sugar beet quality by reducing sugar losses to molasses. Efficient nutrient cycling and improved soil aeration enhance sugar metabolism and accumulation within the beet, thereby increasing sucrose purity and reducing impurities [76]. Studies have shown that soil microbial communities produce extracellular polysaccharides that bind soil particles, enhancing aggregate stability and porosity, which improve water infiltration and hydraulic conductivity [77,78]. Furthermore, enzymatic activity-particularly DHA and Phosphatase- has been linked to improved nutrient availability and enhanced crop productivity, including higher sugar yields [79]. Research also indicates that higher microbial biomass and enzyme activity can enhance nutrient mineralization rates, contributing to better plant nutrient uptake and improved sugar beet quality parameters, including reduced SLM [80]. These findings highlight the interconnected roles of soil biology and physical properties in influencing crop yield and quality.

**Fig 6:**
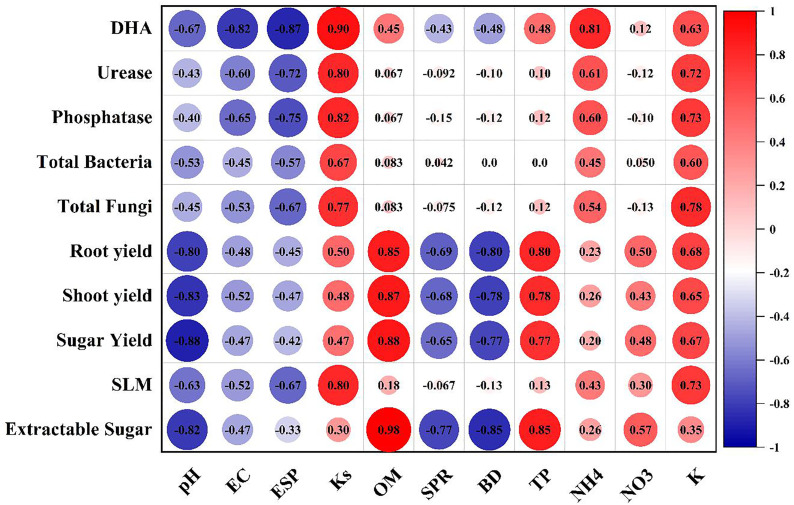
Spearman correlation plot between soil physicochemical parameters and their responses in both soil micro-physiological properties and sugar beet yield characteristics as affected by different K- sources and glauconite extracts after two successful seasons.

The correlation heatmap in (Fig 6) illustrates the relationships between soil chemical properties (pH, EC, ESP, Ks, OM, SPR, BD, TP, NH₄, NO₃, K), and responses of soil enzymatic activities (DHA, urease, phosphatase), microbial populations (total bacteria, total fungi), and sugar beet yield components (root yield, shoot yield, sugar yield, SLM, extractable sugar). The DHA showed a strong correlation with Ks (r = 0.90) and NH_4_ (r = 0.80), suggesting that enhanced microbial respiration and energy metabolism contribute to improved soil structure and nutrient availability. This finding is consistent with previous studies demonstrating that DHA is a reliable indicator of microbial oxidative activity and soil fertility, particularly in soils rich in organic matter [81]. Furthermore, the positive relationship between DHA and Ks indicates that microbial activity may improve soil aggregation and porosity, leading to better water infiltration and movement within the soil profile [82,83]. Urease, phosphatase, TB, and TF showed positive correlations with Ks (r = 0.80, 0.82, 0.67, and 0.77) and K (r = 0.72, 0.73, 0.60, and 0.78). These relationships highlight the role of soil enzymes and microbial populations in nutrient cycling and availability, which are crucial for maintaining soil fertility and promoting plant growth. Urease facilitates nitrogen mineralization by converting urea to ammonium, thereby enhancing nitrogen availability for plant uptake [84–86]. Phosphatase activity, on the other hand, aids in phosphorus mineralization, promoting nutrient availability and uptake by plants [84]. The strong correlations observed with Ks suggest that microbial communities and enzyme activities are essential for maintaining soil structure and hydraulic conductivity, likely through the production of extracellular polysaccharides that stabilize soil aggregates and improve porosity [22]. Organic matter (OM) was found to have significant positive impacts on sugar beet yield components, including Root yield (r = 0.85), Shoot yield (r = 0.87), Sugar yield (r = 0.88), and Extractable Sugar yield (r = 0.98). This strong association underscores the critical role of OM in enhancing soil fertility and productivity by supplying essential nutrients and improving soil physical properties such as aggregation, water retention, and aeration [87,88]. As well as, OM supports microbial activity and enzymatic processes, thereby influencing nutrient cycling and availability, which are crucial for optimal crop growth and yield [33]. However, OM exhibited a minimal impact on Sugar Loss to Molasses (SLM) (r = 0.18), suggesting that factors other than organic content, such as soil salinity or nutrient imbalances, may play a more significant role in influencing sugar quality. This observation aligns with findings from [19], which highlighted the complex interactions between soil properties, nutrient availability, and sugar beet quality parameters.

**Fig 7:**
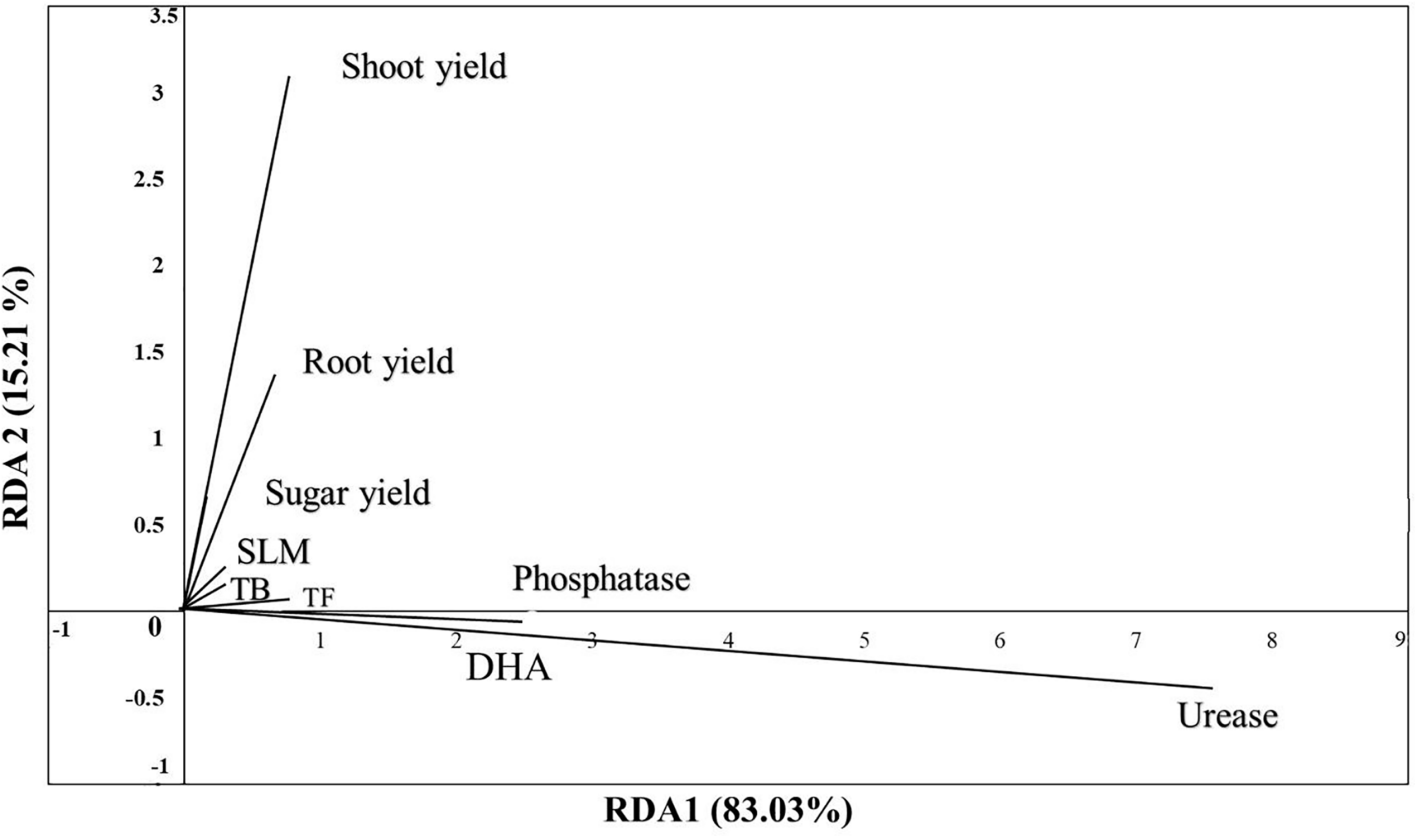
Redundancy Analysis (RDA) of explanatory matrix including soil measured physicochemical properties on a response matrix including soil properties (phosphatase, urease, dehydrogenase “DHA”, total count of bacteria “TB”, and total count of fungi “TF”) and sugar beet yield characteristics (shoot yield, root yield, sugar yield, and sugar losses to molasses” SLM”) as affected by different K- sources and glauconite extracts after two successful seasons of sugar beet plants.

The Redundancy Analysis (RDA) is a powerful multivariate statistical tool used to explore the relationships between matrix variables and matrix responses, making it highly valuable in soil-plant interactions studies. In the present study, RDA biplot in (Fig 7) was employed to assess the influence of soil physicochemical properties on soil enzymatic activities, microbial populations, and sugar beet yield components under different potassium (K) sources and glauconite extracts after two successive growing seasons. The first RDA axis (RDA1) explained 83.03% of the total variance, while the second axis (RDA2) accounted for 15.21%, indicating that the majority of the variation in soil microbial activity and yield characteristics can be attributed to the explanatory variables. The analysis revealed a strong association between shoot yield, root yield, and sugar yield, which were positively aligned along RDA2, suggesting that these yield components were significantly influenced by soil properties. Soil microbial activity, as represented by total bacteria (TB) and total fungi (TF), clustered closely with sugar losses to molasses (SLM), indicating that microbial-driven soil processes could impact sugar beet quality. This aligns with previous studies showing that increased microbial biomass contributes to organic matter decomposition and nutrient mineralization, which in turn affects sugar recovery in beet crops [87–90]. The proximity of microbial biomass to SLM suggests that microbial activity influences the degradation of organic matter and the release of nutrients, affecting sugar accumulation and purity. Among soil enzymatic activities, phosphatase exhibited a moderate association with microbial biomass and yield components, highlighting its role in phosphorus mineralization and nutrient availability. Dehydrogenase activity (DHA) showed a strong correlation with microbial populations and yield parameters, reinforcing its significance as an indicator of microbial oxidative activity and overall soil health [81]. In contrast, urease was positioned distinctly along RDA1 with a negative association with sugar beet yield, suggesting that excessive nitrogen mineralization could have a detrimental impact on sugar accumulation. This is consistent with findings that high nitrogen availability can promote excessive vegetative growth at the expense of sugar partitioning in sugar beet plants [91]. The distribution of soil properties and microbial activity in the RDA plot suggests that different K sources and glauconite extracts significantly influenced soil biochemical properties and their subsequent effects on sugar beet yield. Glauconite-extracted K sources likely enhanced soil enzymatic activities, leading to improved nutrient cycling and root-zone microbial interactions. This is in line with findings by [25], who demonstrated that enhanced microbial activity improves soil aggregation and nutrient retention, thereby creating favorable conditions for plant growth.

Additionally, the observed relationship between organic matter and microbial activity supports previous studies emphasizing the role of organic amendments in boosting microbial biomass and enzyme production, ultimately improving soil fertility and crop productivity [92]. Overall, the RDA analysis underscores the pivotal role of soil enzymatic activities and microbial populations in determining sugar beet yield under varying K sources and glauconite applications. The strong influence of phosphatase and DHA on yield components highlights the importance of maintaining balanced microbial activity for optimal nutrient availability. Meanwhile, the negative association of urease with yield components stresses the need for precise nitrogen management to prevent excessive vegetative growth at the expense of sugar accumulation. These findings emphasize the necessity of integrating soil biological indicators into soil fertility management strategies to enhance sugar beet production, particularly in saline-affected environments.

## 5. Conclusions

- Glauconite-based amendments, particularly foliar-applied extracts, proved effective in alleviating salinity and sodicity constraints in saline soils compared with conventional K₂SO₄ fertilization.
- High-dose glauconite extracts (GE2 and GF2) significantly improved soil physicochemical properties, achieving marked reductions in soil EC (up to 35.11%) and ESP (up to 23.33%), alongside improvements in hydraulic conductivity and organic matter content.
- The fulvic acid extract at 40 mL L ⁻ ¹ (GF2) resulted in the greatest enhancement of soil fertility, notably increasing available K (+132.90%) and stimulating microbial and enzymatic activities, while GE2 was particularly effective in improving micronutrient availability and enzyme function.
- Sugar beet productivity responded positively to glauconite extracts, with GF2 producing substantial increases in root yield (+67.79%), shoot yield (+107.33%), and sugar yield (+92.11%) relative to the control.
- Multivariate analyses (PCA) demonstrated strong positive associations between yield traits and soil available K, organic matter, and microbial activity, whereas elevated pH, EC, and ESP were negatively correlated with crop performance.
- Overall, glauconite extracts—especially GF2 and GE2—represent promising, sustainable alternatives to conventional potassium fertilizers for improving soil quality and sugar beet productivity in salt-affected soils.
- Further research is recommended to evaluate long-term field performance, refine application strategies, and assess the broader impacts of glauconite-based amendments on soil microbial diversity and other cropping systems.

## Supporting information

S1 Data Supplementary data. (XLSX)
